# MRGBMDAT: a multi-relational graph encoder network with bilinear fusion for miRNA-disease association type prediction

**DOI:** 10.1093/bioinformatics/btag534

**Published:** 2026-07-22

**Authors:** Yan Sun, Wenjing Su, Siqi Zhu, Shijia Yan, Xuenan Shi, Junliang Shang, Jin-Xing Liu

**Affiliations:** College of Engineering, Qufu Normal University, Rizhao, Shandong 276826, China; School of Computer Science, Qufu Normal University, Rizhao, Shandong 276826, China; School of Computer Science, Qufu Normal University, Rizhao, Shandong 276826, China; School of Computer Science, Qufu Normal University, Rizhao, Shandong 276826, China; School of Computer Science, Qufu Normal University, Rizhao, Shandong 276826, China; School of Computer Science, Qufu Normal University, Rizhao, Shandong 276826, China; Rizhao-Qufu Normal University Joint Technology Transfer Center, Qufu Normal University, Rizhao, Shandong 276826, China; Shandong Key Laboratory of Wetland Ecology and Biodiversity Conservation in the Lower Yellow River, Qufu, Shandong 273165, China; School of Health and Life Sciences, University of Health and Rehabilitation Sciences, Qingdao, Shandong 266113, China

## Abstract

**Motivation:**

MicroRNAs (miRNAs) are key post-transcriptional regulators involved in diverse biological processes, and their dysregulation is closely associated with the onset and progression of many diseases. Accurate prediction of miRNA-disease association types is therefore essential for understanding disease mechanisms and advancing precision medicine. Although computational methods provide efficient alternatives to wet-lab experiments, existing approaches often focus on binary association prediction, inadequately integrate local semantic dependencies and global topological structures, and suffer from class imbalance.

**Results:**

To address these limitations, we propose MRGBMDAT, a multi-relational graph encoder network with bilinear fusion for miRNA-disease association type prediction. Specifically, a multi-relational graph convolution module with bidirectional cross-attention captures global topological structures, while a local subgraph sampling module extracts local semantic dependencies. A bilinear fusion decoder with element-wise attention jointly models their linear and nonlinear interactions. In addition, an iterative feature similarity-based negative sample selection strategy is introduced to alleviate class imbalance. Experimental results on the HMDD v3.2 dataset demonstrate that MRGBMDAT significantly outperforms five state-of-the-art methods across multiple evaluation metrics, exhibiting strong discriminative power and generalization capability.

**Availability and implementation:**

The source code is publicly available at https://github.com/CDMBlab/MRGBMDAT.

## 1 Introduction

MicroRNAs (miRNAs) are endogenous non-coding RNAs of approximately 22 nucleotides that regulate gene expression at the post-transcriptional level by promoting target mRNA degradation or translational repression. They are involved in numerous biological processes, including cell proliferation, differentiation, apoptosis, and signal transduction (Bartel 2004). Increasing evidence indicates that miRNAs directly influence the initiation and progression of various diseases through the regulation of target genes and signalling pathways (Esteller 2011). For example, miR-21 (He *et al.* 2016) promotes tumour proliferation and invasion by suppressing tumour suppressor genes such as PTEN and PDCD4, whereas the let-7 family inhibits tumour development through the regulation of oncogenes, including RAS and MYC (Zhang *et al.* 2007). In cardiovascular diseases, miR-1 and miR-133 contribute to myocardial hypertrophy and arrhythmia through the regulation of cardiomyocyte differentiation and ion channel activity (Besser *et al.* 2014). In neurodegenerative disorders, dysregulated miR-29 and miR-132 expression is closely associated with pathological protein accumulation and neuroinflammatory responses (Delay *et al.* 2012). Moreover, the same miRNA may exhibit distinct or even opposite expression patterns across different disease contexts or stages, resulting in divergent biological effects through different gene regulatory networks (van Rooij *et al.* 2012). Although experimental approaches provide reliable validation of miRNA functions, they are often labor-intensive, time-consuming, and costly. Therefore, computational methods have emerged as an efficient alternative for identifying candidate miRNA-disease associations and guiding subsequent experimental validation.

Current primary computational methods focus on binary classification prediction tasks that simply determine the presence or absence of an association between a given miRNA and a specific disease, thus failing to characterize the specific functional regulatory types of miRNAs during disease development (Chen *et al.* 2021). Hence, it is highly necessary to shift the research focus to miRNA-disease association type prediction, which can explicitly distinguish different regulatory types to accurately characterize their functional roles in diseases. Chen *et al.* (2015) pioneered this research direction and proposed RBMMMDA based on restricted Boltzmann machines. However, RBMMMDA only utilizes known miRNA-disease associations and ignores similarity information of miRNAs and diseases, resulting in limited predictive performance. To address this issue, Zhang *et al.* (2018) developed NLPMMDA, which integrates multiple similarity networks into a heterogeneous network and employs label propagation for association type prediction. Although its performance is improved, correlations among association types are still neglected. Since association type data can be naturally represented as tensors, several tensor decomposition-based methods have subsequently been proposed. Huang *et al.* (2021) developed TDRC to capture global multi-type association patterns through low-rank tensor decomposition, while Yu *et al.* (2022) proposed TFLP by combining tensor decomposition with label propagation. Ouyang *et al.* (2022) further introduced self-supervised learning into hypergraph non-negative tensor decomposition and proposed SPLDHyperAWNTF, whereas Ma and Ma (2024) developed KBLTDARD, which incorporates biomedical prior knowledge into a Bayesian tensor decomposition framework. Nevertheless, these methods generally rely on relatively dense data, and their performance may be compromised by sparse samples and imbalanced association type distributions.

More recently, graph neural network (GNN)-based methods have been introduced for miRNA-disease association type prediction, yielding encouraging performance. Yan *et al.* (2022) proposed PDMDA, which extracts miRNA and disease representations through fully connected networks and GNNs, respectively, followed by a multi-layer classifier for association type prediction. Zhang *et al.* (2022a) modelled miRNA-disease associations as a signed bipartite network and developed SGNNMD to predict deregulation types. Wang *et al.* (2021) presented NMCMDA, which employs a relational graph convolutional network encoder and a neural multi-relational decoder to predict different association types. Yu *et al.* (2024) proposed MRFGMDA, which utilizes relation-aware message passing on a global heterogeneous network to identify disease-related miRNAs and their association types. However, it mainly focuses on global relational modeling and does not explicitly consider local subgraph-level contextual dependencies or cross-entity interaction enhancement. Zhu *et al.* (2025) proposed SMCLMDA, a statistical meta-path contrastive learning method for identifying multidimensional miRNA-disease relationships. Liu *et al.* (2025) presented DeepMDpred, which combines graph convolution and attention learning for association type prediction. Yu *et al.* (2021) developed mDLinker, which learns latent representations from attributed multi-layer heterogeneous networks and predicts association types for miRNA-disease pairs. These GNN-based methods offer advantages over traditional machine learning and tensor decomposition methods in capturing structural features and modeling complex nonlinear interactions, providing a promising paradigm for miRNA-disease association type prediction. Nevertheless, two challenges remain. First, existing methods still struggle to effectively integrate local semantic dependencies with global topological structures and lack a systematic global-local feature fusion framework. Second, sample distribution imbalance severely limits predictive performance and generalization ability.

To address these challenges, this study presents MRGBMDAT, a multi-relational graph encoder network incorporated with bilinear fusion, tailored for miRNA-disease association type prediction. Specifically, a multi-relational graph convolution module integrated with bidirectional cross-attention is designed to capture global topological structures, while a local subgraph sampling module is adopted to mine local semantic dependencies. A bilinear fusion decoder with element-wise attention is further employed to synergistically model linear and nonlinear correlations between global topological structures and local semantic dependencies. Moreover, an iterative negative sample selection strategy based on feature similarity is introduced to alleviate the class imbalance issue prevalent in datasets. Experimental evaluations on the HMDD v3.2 dataset demonstrate that MRGBMDAT achieves significant performance improvements over five state-of-the-art baseline methods across various evaluation metrics, thus providing a reliable computational framework for miRNA-disease association type prediction.

## 2 Materials and methods

### 2.1 Overview of MRGBMDAT

**Figure 1:**
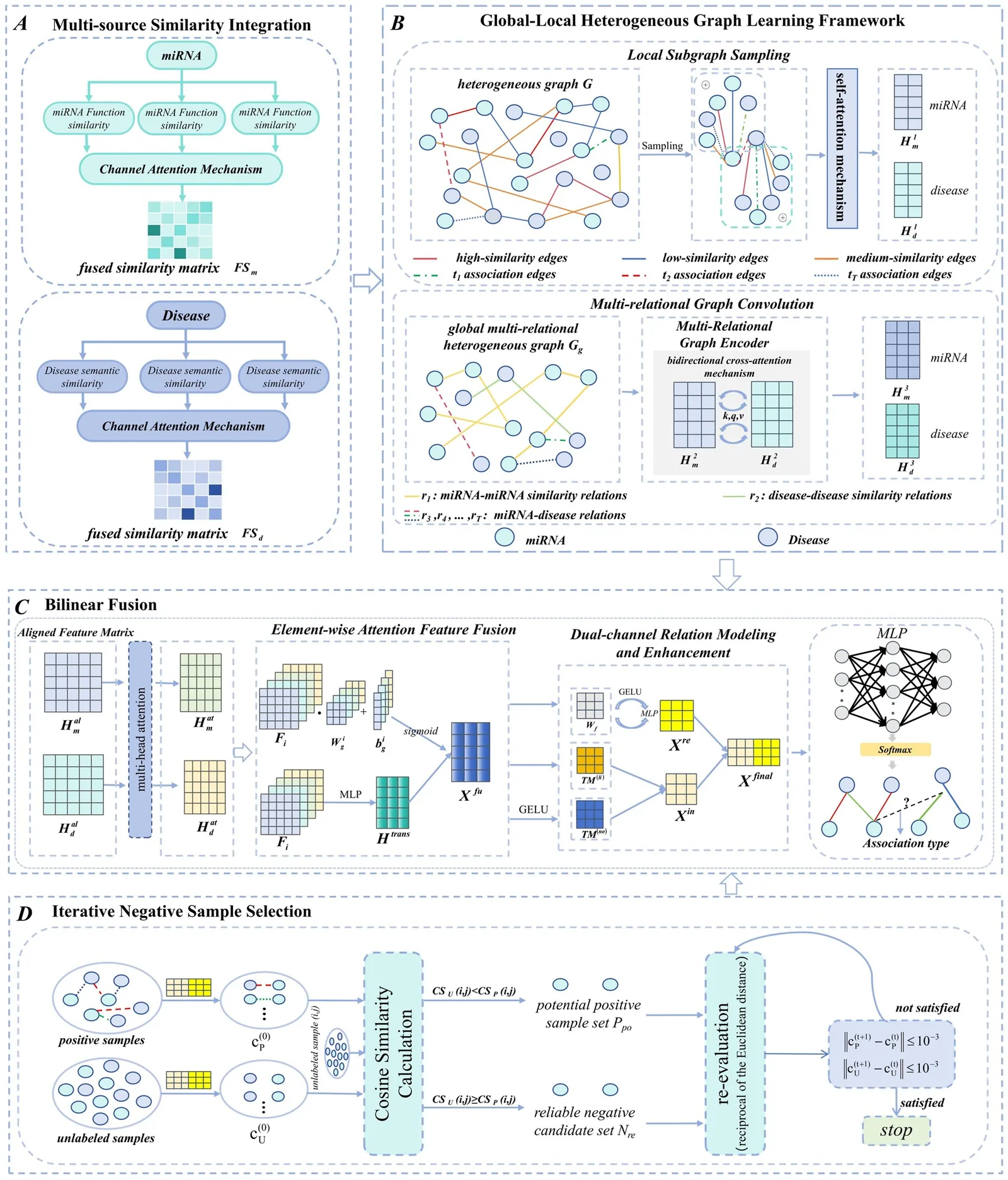
Overview of MRGBMDAT.

MRGBMDAT is specifically designed for miRNA-disease association type prediction, which integrates global topological structures and local semantic dependencies into unified feature representations while alleviating the sample distribution imbalance issue in datasets. As illustrated in Fig. 1, MRGBMDAT comprises four interconnected components: (1A) Multi-source Similarity Integration, (1B) Global-Local Heterogeneous Graph Learning Framework, (1C) Bilinear Fusion, and (1D) Iterative Negative Sample Selection.

### 2.2 MiRNA and disease similarities

Each similarity characterizes miRNA and disease relationships from a distinct biological perspective, providing complementary information for miRNA-disease association type prediction. Therefore, MRGBMDAT integrates miRNA functional similarity, miRNA sequence similarity, disease semantic similarity, target-based disease similarity, and Gaussian interaction profile kernel similarity.

For miRNAs, functional similarity is calculated using the Xiao method (Xiao *et al.* 2018), which combines miRNA-gene interactions with the HumanNet functional network based on the assumption that functionally related genes are regulated by functionally similar miRNAs (Wang *et al.* 2010). Sequence similarity is computed using the Needleman-Wunsch algorithm on miRNA seed regions (Rashed *et al.* 2021). Gaussian interaction profile kernel similarity is derived from known miRNA-disease associations, assuming that miRNAs with similar interaction profiles tend to be associated with similar diseases (Chen *et al.* 2016).

For diseases, semantic similarity is computed from Medical Subject Headings-based directed acyclic graphs (Zhang *et al.* 2022b), while target-based similarity is derived from disease-gene associations by measuring shared molecular targets (Cheng *et al.* 2014). Gaussian interaction profile kernel similarity is similarly calculated from known miRNA-disease associations, assuming that diseases with similar interaction profiles are associated with similar miRNAs.

### 2.3 Multi-source similarity integration

To effectively exploit heterogeneous similarity information and overcome the limitations of traditional integration strategies (e.g. concatenation and averaging), a multi-source similarity integration strategy based on a channel attention mechanism is adopted (Ning *et al.* 2024). Each miRNA and disease similarity is treated as an independent channel, and the attention mechanism is used to adaptively learn its importance weight, thereby generating more informative and robust representations for biological entities while reducing redundancy.

For the *c*-th (c=1,2,3) channel, the corresponding miRNA similarity matrix is denoted as $S_{m}^{c}$, where the entry $S_{m}^{c}(i,j)$ indicates the similarity score between the *i*-th and the *j*-th miRNAs. Global average pooling is applied to $S_{m}^{c}$ to obtain a global descriptor $P_{m}^{c}$,


(1) $$P_{m}^{c}=\frac{1}{n_{m}^{2}}\sum_{i=1}^{n_{m}}\sum_{j=1}^{n_{m}}S_{m}^{c}(i,j),$$

Where $n_{m}$ is the number of miRNAs in the similarity matrix. Analogously, the global descriptor $P_{d}^{c}$ for the disease similarity matrix $S_{d}^{c}$ of the *c*-th channel is also obtained.

Considering that different similarity channels contribute unequally to miRNA feature representations, an attention mechanism is introduced to adaptively weight these channels based on global descriptors. Specifically, a two-layer fully connected attention network is employed to learn the importance weights of the channels,


(2) $$w_{m}^{c}=\delta \left(W_{1}\cdot \sigma \left(W_{2}\cdot P_{m}^{c}\right)\right),$$

Where $W_{1}$ and $W_{2}$ denote learnable parameter matrices, δ(·) corresponds to the SoftMax activation function, and σ(·) denotes the ReLU activation function. Finally, the learned importance weights are element-wise applied to miRNA similarities to perform weighted integration,


(3) $${\text{FS}}_{m}(i,j)=\sum_{c=1}^{3}w_{m}^{c}\cdot S_{m}^{c}(i,j)$$

In the same manner, the procedure is applied to disease similarity matrices to obtain the integrated disease similarity matrix ${\text{FS}}_{d}$.

### 2.4 Global-local heterogeneous graph learning framework

To address the challenge of integrating global topological structures and local semantic dependencies in miRNA-disease networks, a global-local heterogeneous graph learning framework is proposed, consisting of a multi-relational graph convolution module and a local subgraph sampling module. The key distinction between these modules lies in the granularity and scope of the information they capture. The multi-relational graph convolution module, integrated with bidirectional cross-attention, assigns distinct weight matrices and propagation paths to each association type, enabling long-distance information flow along semantically meaningful paths and capturing long-range dependencies as well as global topological structures arising from multi-relational interactions. By maintaining independent message propagation for different association types, the framework preserves relation-specific regulatory information among miRNA-disease pairs. This enables separate learning of relation-specific features, reducing semantic interference across association types. The local subgraph sampling module focuses on fine-grained and functionally cohesive neighborhood patterns to facilitate the extraction of local semantic dependencies. Since miRNAs and diseases with similar biological functions or pathological processes tend to form localized, densely connected modules in heterogeneous biological networks, preserving the neighborhood structure around a target miRNA-disease pair retains relation-specific biological context and functional interaction patterns relevant to different association types. Together, the two modules complement each other by jointly modeling macroscopic relational pathways and microscopic interaction dynamics.

#### 2.4.1 Node feature initialization

To reduce feature dimensionality while retaining original information as much as possible, the integrated similarity matrices of miRNAs and diseases are mapped into a unified feature space via a learnable linear transformation. The obtained features are adopted as the initial node representations in the heterogeneous graph,


(4) $$X=\sigma \left(W\cdot \left(FS_{m}⊕FS_{d}\right)+b\right)$$

Where the operator ⊕ denotes the matrix direct sum, *W* is a learnable weight matrix for linear transformation, and *b* is the bias term.

#### 2.4.2 Local subgraph sampling

The heterogeneous graph is constructed based on the miRNA-disease association matrix and the integrated similarity matrices of miRNAs and diseases. Specifically, miRNA-disease associations are distinguished not only by their existence but also by their specific association types, while similarities between miRNAs and between diseases are divided into three levels, i.e., low, medium, and high. Upon this graph, for each miRNA-disease pair under investigation, one local subgraph centered on the corresponding miRNA and disease nodes is further extracted and employed as the core regions for deep feature learning.

For the heterogeneous graph *G*, miRNA similarity edges and disease similarity edges are denoted as $E_{m}^{\left(l,m,h\right)}$ and $E_{d}^{\left(l,m,h\right)}$, respectively, where l,m, and h represent low-, medium-, and high-similarity edges. The miRNA-disease association edges are denoted as $E_{md}^{\left(t_{1},t_{2},\ldots ,t_{T}\right)}$, where $t_{1}$ represents the first association type and *T* is the total number of association types. Hence, G=(V,E), $V=\{V_{m},V_{d}\}$, and $E=\{E_{m}^{\left(l,m,h\right)},E_{d}^{\left(l,m,h\right)},E_{md}^{\left(t_{1},t_{2},\ldots ,t_{T}\right)}\}$, with $V_{m}$ and $V_{d}$ denoting the sets of miRNA nodes and disease nodes, respectively.

Based on this heterogeneous graph *G*, a local subgraph is extracted for each miRNA-disease pair. For a given miRNA-disease pair $\left(m_{i},d_{j}\right)$, its local subgraph is defined as,


(5) $$G_{ij}=(\{V_{m_{i}},V_{d_{j}}\},\{E_{m_{i}},E_{d_{j}}\})$$

Where $V_{m_{i}}=\{m_{i}\}\cup \{\mathrm{neighbors}(m_{i})\}$ includes the central miRNA node $m_{i}$ and all its neighboring nodes in *G*, and $E_{m_{i}}$ denotes the complete set of edges within $V_{m_{i}}$. Analogously, $V_{d_{j}}$ and $E_{d_{j}}$ denote the corresponding node set and edge set for the disease node $d_{j}$.

In the local contextual environment, different neighboring nodes and connecting edges may convey relation-specific biological signals with varying relevance to distinct miRNA-disease regulatory patterns. To account for such heterogeneity, an attention mechanism is employed to integrate node neighborhood information and multi-semantic edge information, enabling the model to selectively aggregate informative neighbors and association types while effectively capturing multi-level semantic features associated with different regulatory relationships. For the central node *i* in its local subgraph, its neighborhood representation $z_{i}$ is defined as,


(6) $$z_{i}=\sum_{j\in N(i)}\alpha_{ij}\cdot v_{j}$$

Where N(i) denotes the neighborhood set of node *i*, which consists of its direct 1-hop neighbors in the heterogeneous graph. Specifically, neighbors are determined by non-zero association values and node similarities, and $\alpha_{ij}$ is the normalized attention weight, defined as


(7) $$\alpha_{ij}=\frac{e^{\beta_{ij}}}{\sum_{k\in N(i)}e^{\beta_{ik}}}$$

(8) $$\beta_{ij}=\frac{q_{i}^{T}\cdot k_{j}}{\gamma }$$

Where $\beta_{ij}$ is the unnormalized attention weight, and γ is a scaling factor. The query vector $q_{i}$ is directly derived from the feature representation $h_{i}$ of the central node *i*, i.e., $q_{i}=h_{i}$. The key vector $k_{j}$ and the value vector $v_{j}$ are generated by concatenating the neighboring node feature representation $h_{j}$ with the corresponding edge feature representation $e_{ij}$, followed by a multilayer perceptron (MLP), formulated as,


(9) $$k_{j}=v_{j}=\text{MLP}([e_{ij}∥h_{j}])$$

The edge feature representation $e_{ij}$ is obtained through discretized encoding, which maps the edge type information into a vector representation.

Subsequently, the original feature representation $h_{i}$ of the central node *i* is concatenated with the aggregated neighborhood representation $z_{i}$, and a nonlinear transformation is applied via a feed-forward network to obtain the updated feature representation ${\tilde{h}}_{i}$,


(10) $${\tilde{h}}_{i}=\phi (W_{u}\cdot [h_{i}∥z_{i}]+b_{u})$$

Where ϕ(·) denotes the LeakyReLU activation function, $W_{u}$ is a learnable weight matrix for linear transformation, and $b_{u}$ is the bias term.

Finally, the local feature representations of miRNAs and diseases can be written as,


(11) $$H_{m}^{1}=\{{\tilde{h}}_{m_{i}}|m_{i}\in V_{m}\}$$

(12) $$H_{d}^{1}=\{{\tilde{h}}_{d_{j}}|d_{j}\in V_{d}\}$$

#### 2.4.3 Multi-relational graph convolution

Traditional graph convolutional networks typically adopt a unified aggregation strategy for different types of nodes and edges when handling heterogeneous graphs, making it difficult to adequately model interaction heterogeneity and preserve the semantic distinctions among different association types. To address this limitation, a multi-relational graph convolution module integrated with bidirectional cross-attention is employed, which assigns distinct weight matrices and propagation paths to each association type. Consequently, relation-specific biological information can be propagated and transformed independently, enabling the preservation of association-type-specific regulatory semantics while effectively capturing the long-range dependencies and global topological structures of the heterogeneous miRNA-disease network.

Specifically, a thresholding operation is applied to miRNA similarity edges and disease similarity edges to retain only edges with high similarity scores, thereby reducing noise interference and highlighting reliable structural information. For miRNA-disease association edges $E_{md}^{\left(t_{1},t_{2},\ldots ,t_{T}\right)}$, not only the presence of associations is taken into account, but also semantic labels corresponding to different association types are incorporated to enable refined modeling.

Based on this heterogeneous graph, a multi-relational graph convolution network is employed to aggregate neighbor information under different association types. The feature update of node *i* at the *l*-th layer is defined as,


$$h_{i}^{\left(l\right)}=\sigma \left(h_{i}^{\left(l-1\right)}\cdot W_{0}+\sum_{k=1}^{T}\sum_{j\in N^{t_{k}}(i)}\frac{1}{\left|N^{t_{k}}(i)\right|}h_{j}^{\left(l-1\right)}\cdot W_{t_{k}}+b_{0}\right)$$

Where $h_{i}^{\left(l\right)}$ denotes the feature vector of node *i* at the *l*-th layer, $N^{t_{k}}(i)$ represents the neighborhood set of node *i* under association type $t_{k}$, $W_{0}$ is the self-loop weight matrix, $W_{t_{k}}$ is the trainable weight matrix specific to association type $t_{k}$, and $b_{0}$ is the bias vector. The relation-specific transformation matrices $W_{t_{k}}$ enable association-type-specific semantics to be preserved throughout the message propagation process.

Accordingly, the global feature representations of miRNAs and diseases can be written as,


(13) $$H_{m}^{2}=\{h_{m_{i}}^{\left(l\right)}|m_{i}\in V_{m}\}$$

(14) $$H_{d}^{2}=\{h_{d_{j}}^{\left(l\right)}|d_{j}\in V_{d}\}$$

To further capture deep semantic interactions between miRNA and disease nodes, a bidirectional cross-attention mechanism is applied to the global feature representations. Through bidirectional information exchange, the mechanism selectively emphasizes relevant interactions and reinforces relation-specific information learned from different association types, thereby enhancing the discriminative power of the resulting representations.

Specifically, when miRNA nodes are used as queries and disease nodes as keys and values, the global relational feature representations of miRNAs are calculated as,


(15) $$H_{m}^{3}=\delta \left(\frac{H_{m}^{2}\cdot W_{q}\cdot {\left(H_{d}^{2}\cdot W_{k}\right)}^{T}}{\sqrt{d_{k}}}\right)\cdot H_{d}^{2}\cdot W_{v}+H_{m}^{2}$$

Symmetrically, taking disease nodes as queries and miRNA nodes as keys and values, the global relational feature representations of diseases are calculated as,


(16) $$H_{d}^{3}=\delta \left(\frac{H_{d}^{2}\cdot W_{q}\cdot {\left(H_{m}^{2}\cdot W_{k}\right)}^{T}}{\sqrt{d_{k}}}\right)\cdot H_{m}^{2}\cdot W_{v}+H_{d}^{2}$$

Where $W_{q},W_{k}$, and $W_{v}$ are learnable linear projection matrices, and $d_{k}$ denotes the dimensionality of the attention space.

### 2.5 Bilinear fusion

To fuse the global relational feature representations produced by the multi-relational graph convolution module with the local feature representations extracted by the local subgraph sampling module, a bilinear fusion decoder is adopted. This decoder utilizes a parallel dual-channel architecture to model both linear and nonlinear feature interactions, and integrates an attention mechanism to strengthen information interaction across heterogeneous feature sources.

#### 2.5.1 Feature alignment and fusion

For both miRNA and disease entities, the local and global relational feature representations are aligned and concatenated to form unified representations. Specifically, the aligned miRNA features are given by,


(17) $$H_{m}^{al}=[H_{m}^{1}∥H_{m}^{3}]$$

Similarly, the aligned disease features are constructed as,


(18) $$H_{d}^{al}=[H_{d}^{1}∥H_{d}^{3}]$$

Hence, the overall aligned feature matrix is then formed as,


(19) $$X^{al}=[H_{m}^{al}∥H_{d}^{al}]$$

Which integrates both local and global information for all nodes in the heterogeneous graph.

To further enhance the modeling capacity of complex interactions across different feature dimensions and heterogeneous entities, a multi-head attention mechanism is applied to $X^{al}$ for deep feature fusion. This mechanism enables the model to dynamically weight the importance of different feature components and node interactions. The computation of the *i*-th attention head is defined as,


(20) $$X_{i}^{at}=\delta \left(\frac{X^{al}\cdot W_{q}^{i}{\left(X^{al}\cdot W_{k}^{i}\right)}^{T}}{\sqrt{d_{h}/h}}\right)\cdot (X^{al}\cdot W_{v}^{i})$$

Where $W_{q}^{i},W_{k}^{i}$, and $W_{v}^{i}$ are learnable projection matrices for the *i*-th attention head, *h* denotes the total number of attention heads, and $d_{h}$ represents the dimensionality of the hidden features. The outputs of all attention heads are then concatenated to form the overall attention-enhanced feature matrix,


$$X^{at}=\psi (X_{1}^{at}∥X_{2}^{at}∥\ldots ∥X_{h}^{at})$$

Where ψ(·) denotes the concatenation function.

Finally, $X^{at}$ is split along the node dimension to recover the separate representations for miRNAs and diseases, resulting in the attention-enhanced feature representations,


(21) $$X^{at}=[H_{m}^{at}∥H_{d}^{at}]$$

#### 2.5.2 Element-wise attention feature fusion

To quantify the relative importance of each feature source, an element-wise attention feature fusion mechanism is used to compute an attention weight vector for each source,


(22) $$a_{i}=\varphi (W_{g}^{i}\cdot F_{i}+b_{g}^{i})$$

Where $F_{i}\in \{H_{m}^{al},H_{d}^{al},H_{m}^{at},H_{d}^{at}\}$ denotes the *i*-th feature source, $W_{g}^{i}$ and $b_{g}^{i}$ are the learnable weight matrix and bias vector of the attention projection layer for the *i*-th source, and φ(·) denotes the sigmoid activation function.

In parallel, all feature sources are concatenated and fed into a MLP to perform a global nonlinear transformation, yielding fused global contextual information,


(23) $$H^{\mathrm{trans}}=\mathrm{MLP}\left(\psi (H_{m}^{al}∥H_{d}^{al}∥H_{m}^{at}∥H_{d}^{at})\right)$$

Finally, the fused feature representations are obtained by performing element-wise weighted aggregation over all feature sources and incorporating the globally transformed features,


(24) $$X^{fu}=\sum_{F_{i}}\left(a_{i}⊙F_{i}\right)+b⊙H^{\mathrm{trans}}$$

Where $X^{fu}=[H_{m}^{fu}∥H_{d}^{fu}]$, $H_{m}^{fu}$ and $H_{d}^{fu}$ are respective fused miRNA- and disease-side feature representations, ⊙ denotes Hadamard multiplication, and *b* is an additional learnable global gating vector.

#### 2.5.3 Dual-channel relation modeling and enhancement

The bilinear fusion decoder leverages a parallel dual-channel architecture, empowered by two learnable bilinear transformation matrices, to explicitly capture both linear and nonlinear feature interactions, leading to a more comprehensive and expressive representation of the miRNA-disease association space.

The interactive feature representations from both channels are unified as,


(25) $$X^{in}=\underset{\mathrm{nonlinear}}{\underset{︸}{\omega (H_{m}^{fu})\cdot TM^{\left(no\right)}\cdot {\left(\omega (H_{d}^{fu})\right)}^{T}}}+\underset{\mathrm{linear}}{\underset{︸}{H_{m}^{fu}\cdot TM^{\left(li\right)}\cdot {\left(H_{d}^{fu}\right)}^{T}}}$$

Where $TM^{\left(no\right)}$ and $TM^{\left(li\right)}$ are learnable bilinear transformation matrices for the nonlinear and linear channels, respectively, and ω(·) denotes the Gaussian error linear unit activation function.

To further enhance the discriminative capacity, a lightweight relation enhancement module is introduced to capture the global semantic information of miRNA-disease pairs,


(26) $$X^{re}=\text{MLP}(\omega (X^{fu}\cdot W_{f})+X^{fu}\cdot W_{f})$$

Where $X^{re}$ denotes the relation-enhanced feature representations, and $W_{f}$ is a learnable transformation matrix.

Finally, the interactive feature representations and the relation-enhanced feature representations are concatenated to form the final embedding representations,


(27) $$X^{\mathrm{final}}=[X^{in}∥X^{re}]$$

Which serves as the compact and discriminative feature representation for the subsequent miRNA-disease association type prediction.

### 2.6 Prediction and optimization

Building on the final embedding representations $X^{\mathrm{final}}$, the feature vector of a given miRNA-disease pair $\left(m_{i},d_{j}\right)$ is extracted and fed into a classifier to perform the final prediction of miRNA-disease association types. The predicted probability distribution over *T* association types ${\hat{y}}_{\left(i,j\right)}$ is computed as,


(28) $${\hat{y}}_{\left(i,j\right)}=\delta \left(\text{MLP}(X_{\left(i,j\right)}^{\mathrm{final}})\right)$$

To improve prediction performance and alleviate overfitting, MRGBMDAT is optimized using the cross-entropy loss function, defined as,


(29) $$\mathrm{Loss}=-\frac{1}{\left|N\right|}\sum_{(i,j)\in N}\sum_{t=1}^{T}y_{\left(i,j\right)}^{t}\;\text{log}({\hat{y}}_{\left(i,j\right)}^{t})$$

Where *N* denotes the set of miRNA-disease pairs used for training, $y_{\left(i,j\right)}^{t}$ is the ground-truth label indicating whether $\left(m_{i},d_{j}\right)$ belongs to the *t*-th association type.

### 2.7 Iterative negative sample selection

To alleviate the influence of noisy negatives and class imbalance, an iterative negative sample selection strategy was proposed. Specifically, the final embedding representations were utilized to characterize miRNA–disease pairs, and unlabeled samples were iteratively partitioned into reliable negative candidates and potential positive candidates according to their similarities to the positive and unlabeled sample centroids. During each iteration, centroid representations were updated based on the refined sample sets, and the partitioning process was repeated until convergence. The resulting reliable negative samples were subsequently used for model training. Detailed formulations and implementation procedures are provided in Supplementary Section S1.

## 3 Results and discussion

### 3.1 Datasets and evaluation metrics

MRGBMDAT was evaluated on the MCD-up/down dataset derived from HMDD v3.2. After preprocessing and quality control, the dataset contains 12,446 experimentally supported miRNA–disease associations involving 853 miRNAs and 591 diseases. Detailed dataset construction procedures and statistical characteristics are provided in Supplementary Section S2.

To comprehensively assess model performance, we conducted five-fold cross-validation under two complementary settings, namely CVtriplet and CVtype. CVtriplet evaluates the ability to distinguish valid miRNA–disease–type associations from unknown triplets, whereas CVtype focuses on identifying the most probable association type for a given miRNA–disease pair. Performance was measured using AUC, AUPR, and F1-score under CVtriplet, and Top-1 precision, Top-1 recall, and Top-1 F1-score under CVtype. Detailed descriptions of the validation protocols and evaluation metrics are provided in Supplementary Section S3.

### 3.2 Parameter sensitivity analysis

**Figure 2:**
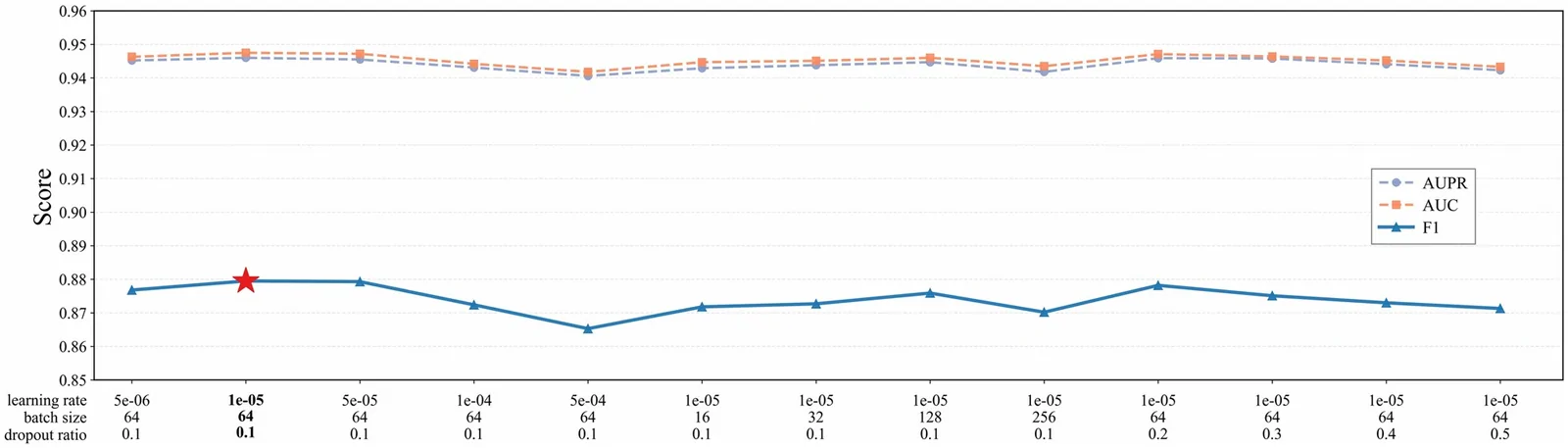
Parameter sensitivity analysis of MRGBMDAT under the CVtriplet setting.

**Figure 3:**
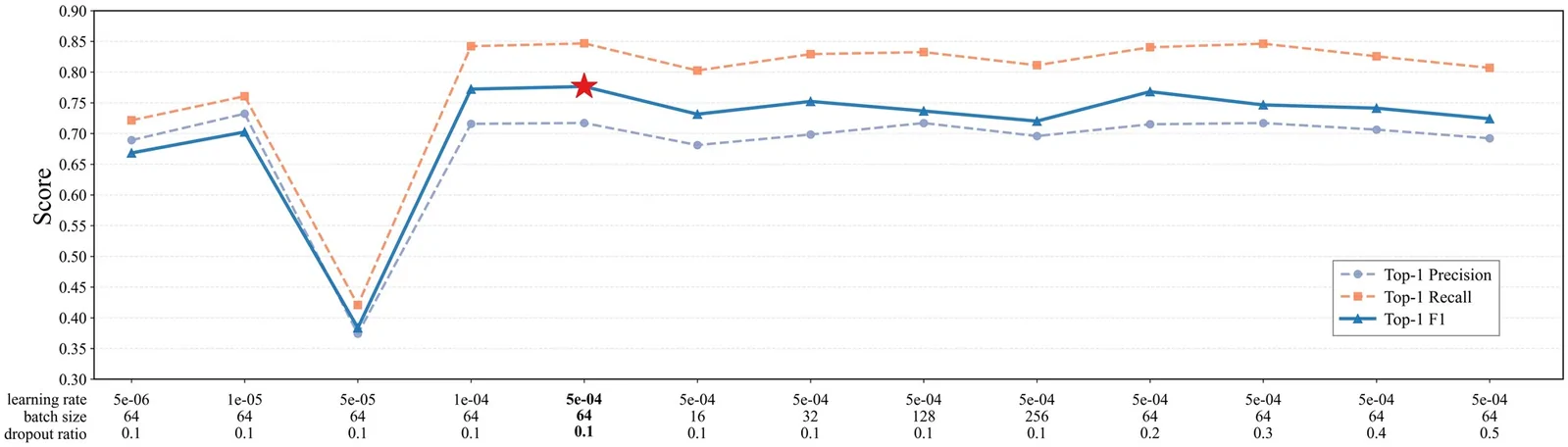
Parameter sensitivity analysis of MRGBMDAT under the CVtype setting.

A comprehensive parameter sensitivity analysis of MRGBMDAT is presented to reveal the effects of key hyperparameters and determine the optimal configuration. The search range of learning rate is determined based on preliminary experiments. Relatively small learning rates starting from $5\times 10^{-6}$ are adopted to ensure stable convergence. Experiments are conducted under both CVtype and CVtriplet settings, with results summarized in Figs. 2 and 3 and Tables 1 and 2, respectively. Three hyperparameters are investigated: learning rate, batch size, and dropout ratio, where one parameter is varied while the other two are fixed. Quantitative results, including mean values and standard deviations over multiple runs, are reported for reliable evaluation.

**Table 1:** Performance comparison of MRGBMDAT across different parameter configurations (learning rate, batch size, and dropout ratio) under the CVtriplet setting.

Configuration	AUPR	AUC	F1
(0.000005, 64, 0.1)	0.9452±0.0032	0.9463±0.0028	0.8768±0.0065
**(0.00001, 64, 0.1)**	**0.9460** ± **0.0021**	**0.9475** ± **0.0024**	**0.8795** ± **0.0022**
(0.00005, 64, 0.1)	0.9455±0.0022	0.9472±0.0017	0.8793±0.0022
(0.0001, 64, 0.1)	0.9431±0.0076	0.9442±0.0061	0.8724±0.0112
(0.0005, 64, 0.1)	0.9406±0.0100	0.9418±0.0089	0.8653±0.0187
(0.00001, 16, 0.1)	0.9429±0.0063	0.9447±0.0052	0.8718±0.0097
(0.00001, 32, 0.1)	0.9438±0.0050	0.9451±0.0045	0.8727±0.0105
(0.00001, 128, 0.1)	0.9447±0.0041	0.9460±0.0035	0.8759±0.0078
(0.00001, 256, 0.1)	0.9418±0.0057	0.9435±0.0049	0.8702±0.0089
(0.00001, 64, 0.2)	0.9459±0.0029	0.9471±0.0026	0.8782±0.0043
(0.00001, 64, 0.3)	0.9458±0.0038	0.9464±0.0023	0.8751±0.0085
(0.00001, 64, 0.4)	0.9441±0.0045	0.9452±0.0039	0.8730±0.0067
(0.00001, 64, 0.5)	0.9423±0.0048	0.9433±0.0034	0.8713±0.0054

**Table 2:** Performance comparison of MRGBMDAT across different parameter configurations (learning rate, batch size, and dropout ratio) under the CVtype setting.

Configuration	Top-1 precision	Top-1 recall	Top-1 F1
(0.000005, 64, 0.1)	0.6892±0.0621	0.7215±0.0584	0.6684±0.0603
(0.00001, 64, 0.1)	**0.7323** ± **0.0575**	0.7608±0.0105	0.7025±0.0224
(0.00005, 64, 0.1)	0.3741±0.3698	0.4208±0.3546	0.3836±0.3754
(0.0001, 64, 0.1)	0.7158±0.0102	0.8421±0.0067	0.7723±0.0081
**(0.0005, 64, 0.1)**	0.7172±0.0090	**0.8468** ± **0.0053**	**0.7766** ± **0.0075**
(0.0005, 16, 0.1)	0.6812±0.0745	0.8026±0.0688	0.7315±0.0719
(0.0005, 32, 0.1)	0.6984±0.0673	0.8293±0.0097	0.7523±0.0420
(0.0005, 128, 0.1)	0.7171±0.0090	0.8326±0.0051	0.7367±0.0062
(0.0005, 256, 0.1)	0.6959±0.0100	0.8112±0.0066	0.7202±0.0083
(0.0005, 64, 0.2)	0.7151±0.0090	0.8405±0.0056	0.7682±0.0081
(0.0005, 64, 0.3)	0.7171±0.0090	0.8463±0.0050	0.7466±0.0074
(0.0005, 64, 0.4)	0.7063±0.0100	0.8257±0.0069	0.7413±0.0088
(0.0005, 64, 0.5)	0.6922±0.0365	0.8069±0.0088	0.7241±0.0095

Under the CVtriplet setting (Fig. 2 and Table 1), MRGBMDAT demonstrates consistently strong and stable performance across parameter configurations, indicating high robustness to hyperparameter variation. A moderate learning rate of 1e-5 yields the most balanced performance among AUC, AUPR, and F1-score. A too small (5e-6) or excessively large (5e-4) learning rate causes slight performance degradation, likely due to slow convergence or suboptimal optimization. Regarding batch size, MRGBMDAT benefits from an increasing batch size, with the value of 64 achieving optimal performance. Performance declines monotonically as dropout increases from 0.1 to 0.5, as excessive regularization hinders discriminative triplet-level representation learning. Overall, the optimal configuration of learning rate, batch size, and dropout ratio under CVtriplet is 1e-5, 64, and 0.1, respectively.

By comparison, under the CVtype setting (Fig. 3 and Table 2), MRGBMDAT displays larger performance variation across hyperparameters, reflecting the greater difficulty of association type prediction. Small learning rates (5e-5) lead to poor and unstable Top-1 precision, recall and F1-score, as MRGBMDAT fails to capture sufficient semantic information for type discrimination. Performance improves noticeably when the learning rate is increased to 1e-4 or 5e-4, especially with a moderate batch size of 64, which helps MRGBMDAT better adapt to cross-type semantic variations. The best Top-1 F1-score, along with the highest recall, is achieved at the optimal configuration: a learning rate of 5e-4, batch size of 64 and dropout ratio of 0.1. Regarding the dropout ratio, although the Top-1 recall exhibits a slight fluctuation at 0.3, the overall performance including the F1-score follows a general declining trend as dropout increases from 0.1 to 0.5. This suggests that excessive regularization eventually suppresses informative features required for accurate type-level discrimination.

In summary, MRGBMDAT shows strong robustness to hyperparameter changes under the CVtriplet setting, while it is more sensitive and requires a relatively large learning rate under the CVtype setting. Based on comprehensive evaluation across both cross-validation settings, a learning rate of 5e-4, batch size of 64, and dropout ratio of 0.1 deliver the most reliable and balanced performance. This parameter configuration is adopted for all subsequent experiments.

### 3.3 Comparison with state-of-the-art methods

To evaluate the performance of MRGBMDAT, comprehensive comparative experiments were performed with five representative state-of-the-art methods for miRNA-disease association type prediction, including TDRC (Huang *et al.* 2021), TFLP (Yu *et al.* 2022), SPLDHyperAWNTF (Ouyang *et al.* 2022), NMCMDA (Wang *et al.* 2021), and MRFGMDA (Yu *et al.* 2024). All baseline methods were evaluated under the same experimental settings and data division strategies to guarantee a fair and objective comparison.

**Figure 4:**
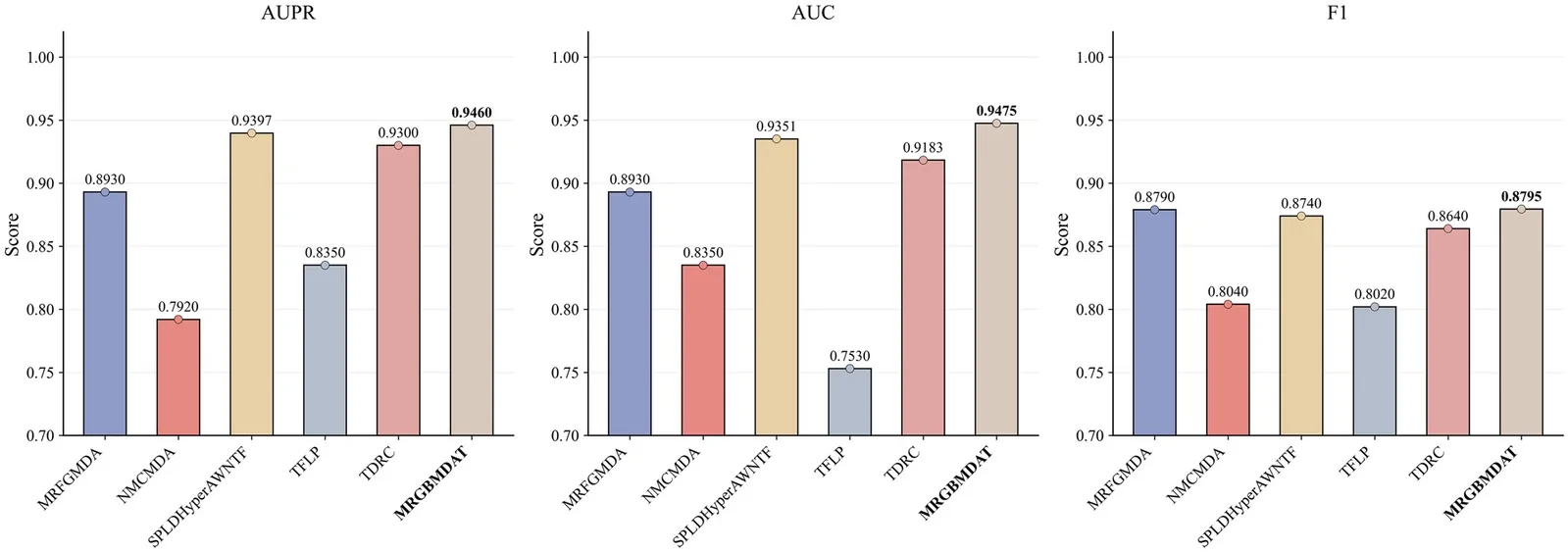
Performance comparison of different methods under CVtriplet settings.

Under the CVtriplet setting, the task is constructed as a binary miRNA-disease association prediction problem (Fig. 4). MRGBMDAT achieves the highest AUPR and AUC values among all compared methods, reaching 0.9460 and 0.9475 respectively. Such results demonstrate its superior ranking ability and robustness under class-imbalanced scenarios. The performance improvements over competitive baselines further verify the effectiveness of MRGBMDAT. For instance, SPLDHyperAWNTF obtains an AUPR of 0.9397 and an AUC of 0.9351, while TDRC yields an AUPR of 0.9300 and an AUC of 0.9183. In comparison, MRGBMDAT exhibits stronger capability in identifying true miRNA-disease associations across the full ranking list. In terms of F1-score, MRGBMDAT achieves a value of 0.8795, which is comparable to the best baseline MRFGMDA and obviously outperforms NMCMDA and TFLP. These results illustrate that MRGBMDAT maintains a good balance between precision and recall, instead of over-optimizing only a single metric.

**Figure 5:**
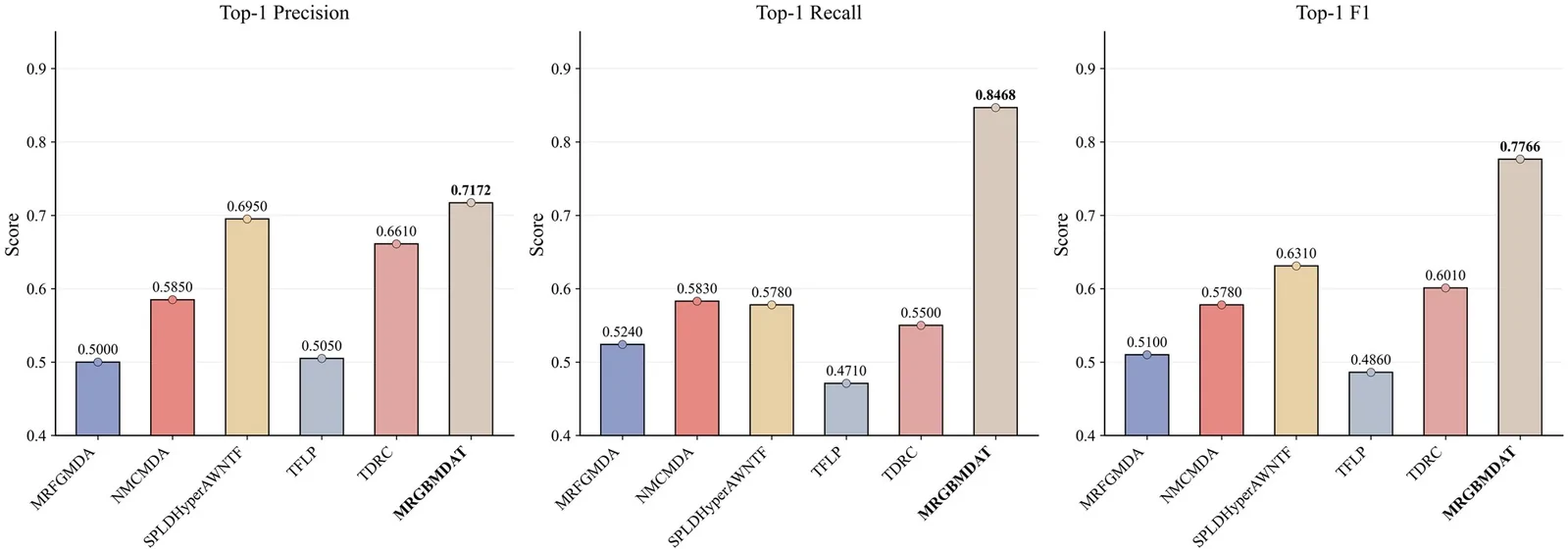
Performance comparison of different methods under CVtype settings.

Under the CVtype setting, the task focuses on identifying the specific association type for each miRNA-disease pair, which is more challenging than conventional binary prediction. As shown in Fig. 5, MRGBMDAT achieves a Top-1 recall of 0.8468, significantly outperforming all competing methods. This result demonstrates its strong ability to recover the true association type among multiple candidates, which is critical for association type prediction. In terms of Top-1 precision, MRGBMDAT reaches 0.7172 and surpasses all compared baselines. Moreover, MRGBMDAT achieves a Top-1 F1-score of 0.7766, which ranks first and is considerably higher than that of the second-best method. Other compared methods suffer from an obvious imbalance between precision and recall, whereas MRGBMDAT improves both metrics simultaneously. Overall, MRGBMDAT is well suited for miRNA-disease association type prediction.

### 3.4 Ablation studies

To investigate the contribution of each core component in MRGBMDAT, ablation experiments are performed under both CVtype and CVtriplet settings through individual removal of the multi-relational graph convolution module (MGC), bidirectional cross-attention mechanism (BCA), bilinear fusion decoder (BFD), and iterative negative sample selection strategy (INS).

**Figure 6:**
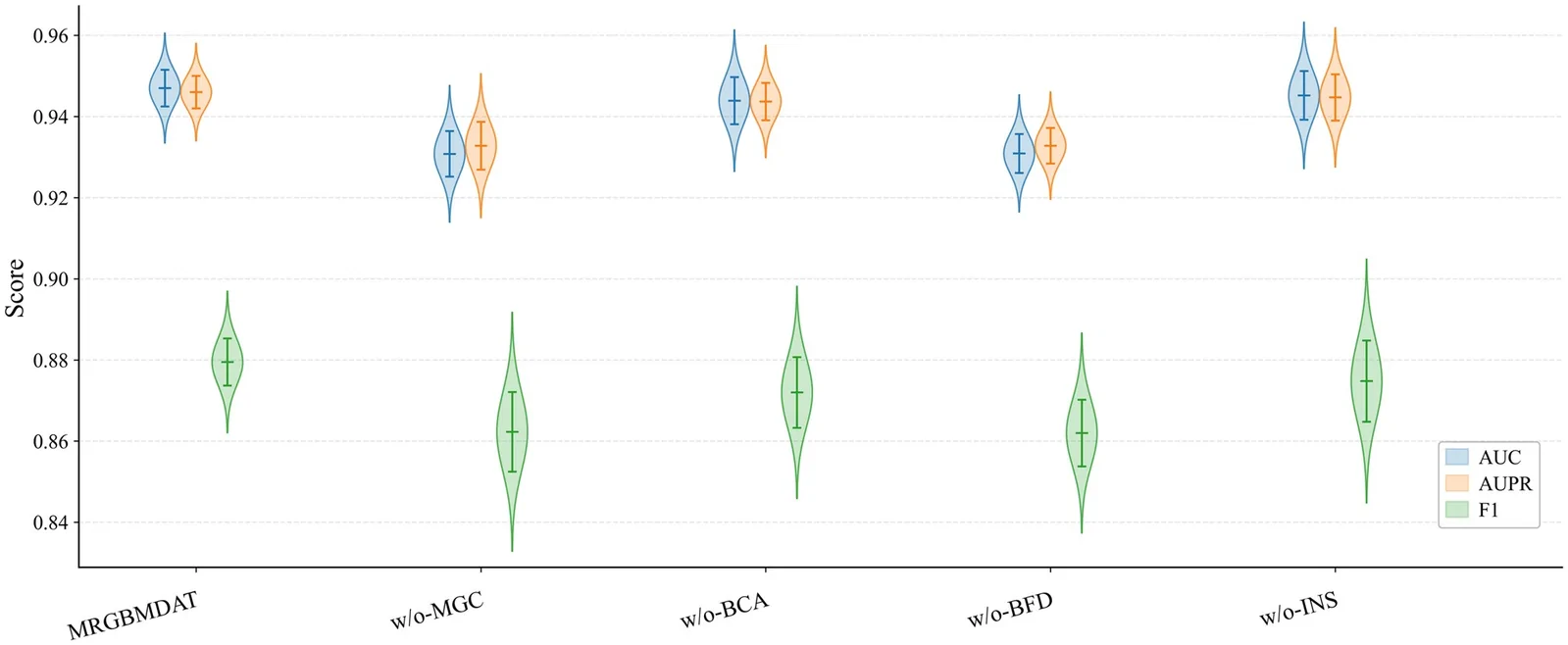
Results of ablation experiments under CVtriplet settings.

Under the CVtriplet setting, MRGBMDAT achieves optimal baseline performance with an AUPR of 0.9460, an AUC of 0.9475, and an F1-score of 0.8795, as presented in Fig. 6. Consistent performance degradation across all evaluation metrics is observed following individual ablation of each core module, which confirms the non-redundant and synergistic design of MRGBMDAT. Specifically, ablation of the MGC module (w/o MGC) results in an AUPR of 0.9324, an AUC of 0.9311, and an F1-score of 0.8615, corresponding to relative decreases of 1.44%, 1.73%, and 2.05%, respectively. These results highlight the efficacy of the multi-relational graph convolution module in capturing and preserving global topological structures. For the bidirectional cross-attention mechanism, ablation of BCA (w/o BCA) reduces the AUPR, AUC, and F1-score to 0.9436, 0.9438, and 0.8715, respectively, demonstrating its critical role in optimizing the integration of heterogeneous biological features. Furthermore, ablation of the BFD module (w/o BFD) leads to a marked performance drop, with AUPR decreasing to 0.9326, AUC to 0.9312, and F1-score to 0.8609. This finding verifies that the bilinear fusion decoder is indispensable for synergistically modeling the linear and nonlinear relationships between global topological structures and local semantic dependencies. Finally, ablation of the INS strategy (w/o INS) yields an AUPR of 0.9448 and an AUC of 0.9453, validating that the feature similarity-based iterative negative sample selection strategy provides a critical safeguard against inherent class imbalance in the dataset.

**Figure 7:**
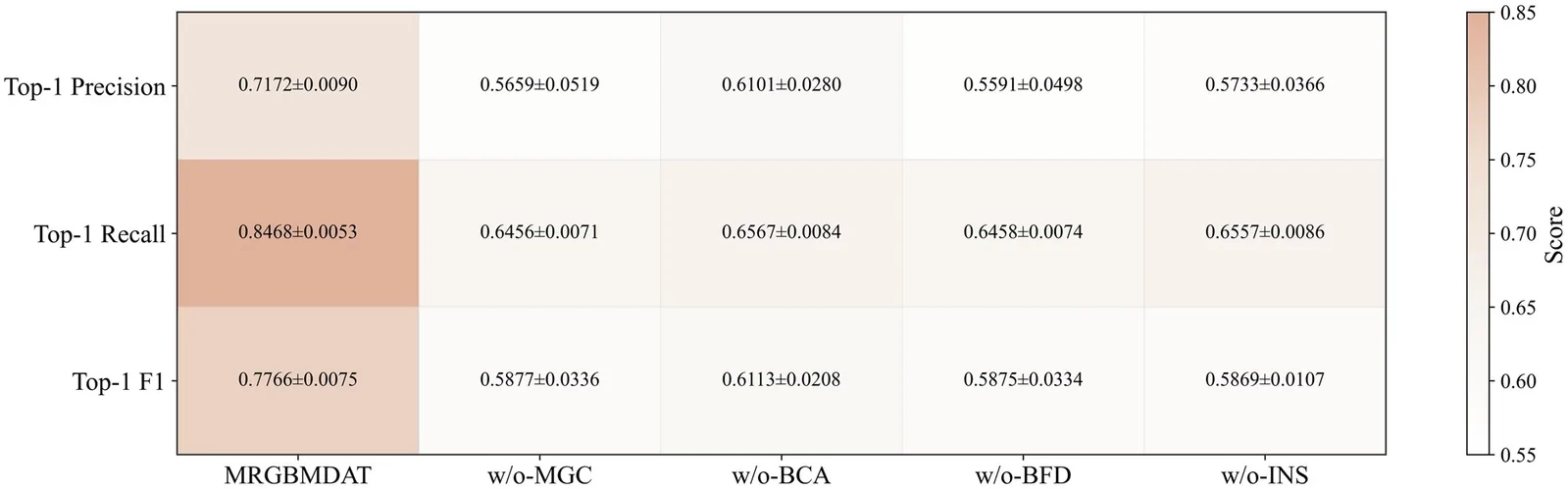
Results of ablation experiments under CVtype settings.

Under the CVtype setting, MRGBMDAT yields robust baseline performance, with a Top-1 precision of 0.7172, a Top-1 recall of 0.8468, and a Top-1 F1-score of 0.7766, as documented in Fig. 7. More pronounced performance degradation is observed across all metrics in association type prediction, which emphasizes the reliance of MRGBMDAT on the integrated design of all core components to resolve complex miRNA regulatory patterns. The most significant performance decline is induced by ablation of the MGC module (w/o MGC), with Top-1 precision, recall, and F1-score decreasing to 0.5659, 0.6456, and 0.5877, respectively. These results confirm that global topological structure modeling is a core prerequisite for accurate association type discrimination. Similarly, ablation of the BFD module (w/o BFD) leads to a sharp performance decline, with Top-1 precision decreasing to 0.5591 and Top-1 F1-score to 0.5875. This finding identifies the BFD as a key component for enhancing the discriminative decoding capability of the framework. For the bidirectional cross-attention mechanism, ablation of BCA (w/o BCA) reduces Top-1 precision to 0.6101 and Top-1 recall to 0.6567, demonstrating the critical role of this mechanism in capturing cross-modal feature correlations. Lastly, ablation of the INS strategy (w/o INS) yields a Top-1 precision of 0.5733 and a Top-1 F1-score of 0.5869, validating that mitigation of inherent class imbalance is fundamental to accurate miRNA-disease association type prediction. Collectively, these ablation results confirm that all core components of MRGBMDAT are indispensable for optimal predictive performance, while also revealing their distinct functional contributions. The multi-relational graph convolution module captures global relational semantics, the bidirectional cross-attention mechanism strengthens semantic interactions between miRNAs and diseases, the bilinear fusion decoder integrates complementary global and local information, and the iterative negative sample selection strategy improves robustness against class imbalance. Their synergistic effects collectively contribute to the effectiveness of the proposed framework.

### 3.5 Case study

To further evaluate the biological plausibility and reliability of MRGBMDAT in predicting miRNA-disease association types, two case studies were performed on breast neoplasms and melanoma, respectively. For each disease, all candidate miRNA-type pairs are ranked according to their prediction scores, and the top 20 highest-scoring combinations are selected for verification. These predictions are then systematically validated against experimentally supported evidence retrieved from the PubMed literature, with a focus on the consistency of regulatory directions and the relevance of biological functions.

For breast neoplasms, the MCD-up/down dataset was adopted to predict the association types, with the top 20 predictions summarized in Table 3. Notably, all predicted association types are fully supported by experimental evidence retrieved from the PubMed literature, which strongly validates the biological plausibility and reliability of the model outputs. For instance, hsa-mir-21 is predicted to be up-regulated, consistent with its well-characterized role as an oncogenic miRNA that drives malignant transformation in breast cancer by targeting PDCD4 (Iorio *et al.* 2005). In contrast, hsa-mir-146a is accurately inferred to be down-regulated, in accordance with its known function in suppressing cell migration and invasion via the ROCK1 signalling pathway (Garcia *et al.* 2011). As a prototypical tumour-suppressive miRNA often silenced in breast cancer, hsa-mir-145 is correctly classified as down-regulated, matching its documented role in restraining tumour cell proliferation through the inactivation of RTK signalling (Gartel and Kandel 2006). Meanwhile, hsa-mir-142 and hsa-mir-150 are reasonably assigned to the other regulatory pattern, supported by their context-dependent functions in immune regulation and remodeling of the tumour microenvironment (Sakurai *et al.* 2015). Collectively, these results illustrate that MRGBMDAT yields biologically consistent and experimentally verifiable predictions of miRNA-disease association types.

**Table 3:** Top 20 predicted miRNA-disease association types related to breast neoplasms.

miRNAs	Types	PMID
hsa-mir-21	up-regulation	16103053
hsa-mir-146a	down-regulation	21472990
hsa-mir-155	up-regulation	16103053
hsa-mir-126	down-regulation	22524830
hsa-mir-223	up-regulation	34953047
hsa-mir-34a	up-regulation	29189128
hsa-mir-29a	down-regulation	28222663
hsa-mir-17	down-regulation	22524830
hsa-mir-145	down-regulation	16466964
hsa-mir-221	down-regulation	24924200
hsa-mir-150	other	25907662
hsa-mir-210	down-regulation	22315424
hsa-mir-142	other	25406066
hsa-mir-222	down-regulation	24924200
hsa-mir-143	up-regulation	26618772
hsa-mir-19a	up-regulation	29189128
hsa-mir-20a	up-regulation	30092355
hsa-mir-122	other	25078559
hsa-mir-15a	up-regulation	29189128
hsa-mir-31	up-regulation	22964023

For melanoma, the same MCD-up/down dataset was employed to assess the generalization ability of MRGBMDAT. The top 20 predicted miRNA-disease association types are summarized in Table 4. Similarly, all predicted association types are fully supported by experimental evidence retrieved from the PubMed literature. For instance, hsa-mir-221 is predicted to be up-regulated, consistent with its well-documented role in driving the transition from a proliferative to an invasive phenotype by downregulating p27Kip1 (Mattia *et al.* 2011). In contrast, hsa-mir-15a is accurately inferred to be down-regulated, in accordance with its established tumour-suppressive function in targeting CDCA4 to restrain aberrant cell cycle progression (Poliseno *et al.* 2012). Meanwhile, hsa-mir-29a is correctly classified as up-regulated, matching its reported involvement in the polarization of tumour-associated macrophages and the establishment of an immunosuppressive tumour microenvironment (Schmitt *et al.* 2012). Furthermore, hsa-mir-34a is reasonably identified as up-regulated in the context of specific regulatory networks, consistent with its complex role in modulating melanoma cell proliferation and sensitivity to therapy (Yang and Wei 2011). Collectively, these results demonstrate that MRGBMDAT serves as a powerful and robust framework for identifying functionally meaningful miRNA-disease association types across diverse cancer types.

**Table 4:** Top 20 predicted miRNA-disease association types related to melanoma.

miRNAs	Types	PMID
hsa-mir-146a	down-regulation	20529253
hsa-mir-21	up-regulation	21509659
hsa-mir-155	down-regulation	19578755
hsa-mir-34a	up-regulation	21509659
hsa-mir-126	other	23217102
hsa-mir-223	up-regulation	28973277
hsa-mir-29a	up-regulation	23245396
hsa-mir-221	up-regulation	21711453
hsa-mir-145	down-regulation	21509659
hsa-mir-17	up-regulation	21509659
hsa-mir-150	other	25054912
hsa-mir-210	up-regulation	19830692
hsa-mir-142	up-regulation	20529253
hsa-mir-222	up-regulation	21711453
hsa-mir-143	other	19639204
hsa-mir-19a	other	24920276
hsa-mir-20a	down-regulation	20529253
hsa-mir-122	down-regulation	19578755
hsa-mir-31	other	21543894
hsa-mir-15a	down-regulation	22551973

## 4 Conclusion

MiRNAs are extensively involved in essential biological processes and exhibit highly heterogeneous functional patterns. The same miRNA may display opposite expression patterns, such as up-regulation or down-regulation, across different disease contexts, posing challenges for elucidating disease mechanisms and developing precise therapeutic strategies. To address this issue, we proposed MRGBMDAT, a multi-relational graph encoder network with bilinear fusion, for accurate miRNA-disease association type prediction. The core innovations of MRGBMDAT are threefold. First, a multi-relational graph convolution module integrated with bidirectional cross-attention facilitates long-distance information propagation and captures global topological structures critical for system-level regulatory networks. Second, a bilinear fusion decoder with element-wise attention models both linear and nonlinear relationships between global topological features and local semantic dependencies, thereby enhancing prediction discrimination. Third, an iterative feature similarity-based negative sample selection strategy adaptively identifies high-quality negative samples, alleviating class imbalance while improving training efficiency and generalization. Experimental results on the HMDD v3.2 dataset demonstrate that MRGBMDAT significantly outperforms five state-of-the-art baseline methods across multiple evaluation metrics, exhibiting strong discriminative power and generalization capability for miRNA-disease association type prediction.

Although MRGBMDAT achieves robust predictive performance, challenges remain in computational efficiency and mechanistic interpretability. The multi-hop sampling strategy and complex attention mechanism introduce computational overhead when processing large-scale multi-omics biological networks, limiting scalability in practical applications. Future work will focus on integrating multi-omics data to construct more informative heterogeneous graphs and developing more efficient graph learning algorithms to further improve scalability, robustness, and practical applicability in miRNA-disease association type prediction.

## Author contributions

Yan Sun (Methodology [Equal], Writing—original draft [Equal]), Wenjing Su (Formal analysis [Equal], Writing—review & editing [Equal]), Siqi Zhu (Visualization [Equal]), Shijia Yan (Investigation [Equal]), Xuenan Shi (Data curation [Equal]), Junliang Shang (Funding acquisition [Equal], Supervision [Equal]), and Jin-Xing Liu (Funding acquisition [Equal], Supervision [Equal])

## Supplementary Material

btag534_Supplementary_Data

## Data Availability

The datasets used in this study (HMDD v3.2) are available at https://www.cuilab.cn/hmdd. The implementation code of MRGBMDAT is publicly available at https://github.com/CDMBlab/MRGBMDAT.
