# Supplementary material for: MRGBMDAT: a multi-relational graph encoder network with bilinear fusion for miRNA-disease association type prediction
Source: Bioinformatics. 2026 Jul 22;42(8):btag534. doi: 10.1093/bioinformatics/btag534 (PMC13447018; doi:10.1093/bioinformatics/btag534)
Supplement: Supplementary file 1 [file btag534_supplementary_data.pdf]

Supplementary Materials

Yan Sun,[1] Wenjing Su,[2] Siqi Zhu,[2] Shijia Yan,[2] Xuenan Shi,[2] Junliang Shang [2,3,4,*] and Jin-Xing Liu[5,*]

[1]College of Engineering, Qufu Normal University, Rizhao, 276826, Shandong, China,

[2]School of Computer Science, Qufu Normal University, Rizhao, 276826, Shandong, China,

[3]Rizhao-Qufu Normal University Joint Technology Transfer Center, Qufu Normal University, Rizhao, 276826, Shandong, China,

[4]Shandong Key Laboratory of Wetland Ecology and Biodiversity Conservation in the Lower Yellow River, Qufu, 273165, Shandong, China,

[5]School of Health and Life Sciences, University of Health and Rehabilitation Sciences, Qingdao, 266113, Shandong, China

*To whom correspondence should be addressed. Email:shangjunliang110@163.com

Section S1. Detailed Implementation of Iterative Negative Sample Selection

In biological association type prediction, experimentally validated positive samples are often scarce, while most unlabeled samples may correspond to either true negatives or undiscovered positive associations. Directly treating all unlabeled samples as negatives introduces substantial noise and degrades model generalization. To address class imbalance in miRNA-disease association type prediction and ensure biologically plausible negative selection, a feature similarity-based iterative negative sample selection strategy is proposed, which leverages the final embedding representations to iteratively identify high-confidence negative samples by comparing the feature similarity of unlabeled samples with the centroids of positive and negative classes.

Specifically, let P and U denote the sets of positive samples and unlabeled samples, respectively. The initial centroid feature vector for any sample set S is defined as,

$$c_S^{(0)} = \frac{1}{|S|} \sum_{(i,j)\in S} X_{(i,j)}^{final}$$

where the initial centroid feature vectors of the positive and unlabeled samples $c_P^{(0)}$, $c_U^{(0)}$ are obtained by substituting $S = P$ and $S = U$, respectively. For each unlabeled sample $(i,j) \in U$, the cosine similarities $CS_P(i,j)$, $CS_U(i,j)$ between its final embedding representation $X_{(i,j)}^{final}$ and the two initial centroids $c_P^{(0)}$ and $c_U^{(0)}$ are computed to quantify its proximity to the positive and unlabeled sets,

$$CS_S(i,j) = \frac{\left(X_{(i,j)}^{final}\right)^{\mathrm{T}} c_S^{(0)}}{\| X_{(i,j)}^{final} \| \cdot \| c_S^{(0)} \| + \epsilon}$$

where ϵ is a small constant added to avoid numerical instability caused by zero vectors, and S takes values of P and U to yield $CS_P(i,j)$ and $CS_U(i,j)$, respectively. If $CS_U(i,j) \geq CS_P(i,j)$, the unlabeled sample (i,j) is considered to be closer to the unlabeled set centroid and is assigned to the reliable negative candidate set N_{re}; otherwise, it is assigned to the potential positive sample set P_{po}.

To further improve the quality of sample partitioning, an iterative refinement strategy is adopted by updating the centroids using the newly partitioned sets,

$$c_P^{(t)} = \frac{1}{|P \cup P_{po}(t)|} \sum_{(i,j)\in P\cup P_{po}(t)} X_{(i,j)}^{final}$$

$$c_U^{(t)} = \frac{1}{|N_{re}(t)|} \sum_{(i,j)\in N_{re}(t)} X_{(i,j)}^{final}$$

where $c_P^{(t)}$ and $c_U^{(t)}$ denote the updated centroids for the refined positive and negative candidate sets at iteration t, respectively.

To provide an alternative similarity perspective and enhance the robustness of selection, the reciprocal of the Euclidean distance is further employed to re-evaluate the similarity between each embedding representation and the updated centroids, and the samples are re-partitioned accordingly,

$$ES_S(i,j) = \frac{1}{\| X_{(i,j)}^{final} - c_S^{(t)} \| + 1}$$

where c takes values of P and U to yield the Euclidean distances $ES_P(i,j)$ and $ES_U(i,j)$, respectively. The unlabeled samples are repeatedly reclassified, and the centroid vectors are continuously updated in an iterative manner until the stopping criteria are satisfied,

$$\left\| c_S^{(t+1)} - c_S^{(t)} \right\| \le 10^{-3}$$

where $S \in \{P,U\}$. This ensures that the centroids stabilize and the negative sample selection converges to a biologically plausible and statistically robust solution.

Section S2. Dataset Description

The Human MicroRNA Disease Database (HMDD) is a widely used benchmark resource that manually curates experimentally validated associations between miRNAs and diseases. HMDD v3.2 further annotates miRNA–disease associations according to different expression patterns, including upregulation, downregulation, and other expression changes.

In this study, we adopted the MCD-up/down dataset derived from HMDD v3.2. To ensure data quality and reliability, duplicated records were removed and only experimentally supported associations were retained. After preprocessing, the final dataset contains 12,446 high-confidence miRNA–disease associations involving 853 miRNAs and 591 diseases.

The dataset explicitly records association types. Specifically, it contains 1,511 upregulation associations, 1,627 downregulation associations, and 9,308 associations belonging to other regulatory categories. These annotations provide a suitable benchmark for miRNA–disease association type prediction.

Table S1. Statistics of the MCD-up/down dataset

Category	Number
miRNAs	853
Diseases	591
Upregulation associations	1511
Downregulation associations	1627
Other associations	9308
Total associations	12446

Section S3. Evaluation Metrics

Different evaluation metrics are adopted for different cross-validation settings to ensure that model performance is assessed in a manner consistent with the corresponding prediction tasks. In this study, two cross-validation settings are employed to systematically evaluate the performance of MRGBMDAT.

Under the CVtriplet setting, all validated miRNA-disease-type triplets as positive samples are randomly split into five subsets. In each fold, one positive subset and an equal number of unknown triplets as negatives form the test set, while the rest positives and equivalent negatives constitute the training set, with no overlap between training and test data. As a binary classification task targeting imbalanced data, the area under the receiver operating characteristic curve (AUC), the area under the precision-recall curve (AUPR), and F1-score are adopted to evaluate ranking performance, where AUPR is highlighted due to the severe class imbalance.

Let TP, FP, TN, FN denote true positives, false positives, true negatives and false negatives, respectively. N_{pos} and N_{neg} represent the total numbers of positive and negative triplets; N_{correct} is the count of positive-negative sample pairs where positive samples yield higher prediction scores, and N_{tie} denotes pairs with identical predicted scores. The calculation formulas of evaluation metrics are defined as follows:

$$\text{Precision} = \frac{TP}{TP+FP},$$

$$\text{Recall} = \frac{TP}{TP+FN},$$

$$\text{F1} = 2\times\frac{\text{Precision}\times\text{Recall}}{\text{Precision}+\text{Recall}},$$

$$\text{AUC} = \frac{N_{\text{correct}}+0.5N_{\text{tie}}}{N_{\text{pos}}\times N_{\text{neg}}},$$

$$\text{AUPR} = \int_0^1 \text{Precision}(\text{Recall})\, d(\text{Recall}).$$

Under the CVtype setting, all miRNA-disease pairs with at least one known association type are randomly divided into five subsets. In each fold of five-fold cross-validation, one subset serves as the test set and the others as the training set. For each test miRNA-disease pair, candidate association types are ranked by predicted scores, and the top-scoring type is taken as the prediction. This setting aims to identify the most likely association type, so Top-1 precision, Top-1 recall, and Top-1 F1-score (Top-1 F1) are used to quantify type-level prediction performance.

Suppose there are K distinct association types. TP_k, FP_k and FN_k correspond to true positives, false positives and false negatives of the k-th

association type under Top-1 prediction. Macro-averaged Top-1 evaluation metrics are calculated via the following formulas:

$$\text{Top-1 Precision}_k = \frac{TP_k}{TP_k + FP_k},$$

$$\text{Top-1 Recall}_k = \frac{TP_k}{TP_k + FN_k},$$

$$\text{Top-1 F1}_k = 2 \times \frac{\text{Top-1 Precision}_k \times \text{Top-1 Recall}_k}{\text{Top-1 Precision}_k + \text{Top-1 Recall}_k},$$

$$\text{Top-1 Precision} = \frac{1}{K}\sum_{k=1}^{K} \text{Top-1 Precision}_k,$$

$$\text{Top-1 Recall} = \frac{1}{K}\sum_{k=1}^{K} \text{Top-1 Recall}_k,$$

$$\text{Top-1 F1} = \frac{1}{K}\sum_{k=1}^{K} \text{Top-1 F1}_k.$$
